# A Thirty‐Year Gap: Reviving All‐Nitride Glasses as Processable, High‐Performance Materials

**DOI:** 10.1002/advs.77963

**Published:** 2026-09-27

**Authors:** Alexis Duval, Tor Grande, Lothar Wondraczek

**Affiliations:** ^1^ Otto Schott Institute of Materials Research Friedrich Schiller University Jena Jena Germany; ^2^ Department of Materials Science and Engineering NTNU Norwegian University of Science and Technology Trondheim Norway; ^3^ Center for Energy and Environmental Chemistry (CEEC) Friedrich Schiller University Jena Jena Germany

**Keywords:** CALPHAD, mechanics, modeling, nitride glasses, optics, structure

## Abstract

This study revisits oxygen‐free, all‐nitride glasses as a distinct class of high‐performance materials, originally reported three decades ago by Grande, Holloway, McMillan, and Angell. A novel glass family at the time, such glass systems have remained unreplicated, largely due to the highly specialized experimental conditions required for their synthesis. With contemporary advances, such constraints are no longer prohibitive. Rather, in light of the persistent challenges in enhancing the performance of conventional glass systems, all‐nitride glasses are reconsidered herein as a potentially viable class of processable materials that combine the formability of glasses with ceramic‐level performance–without compromising optical transparency. Building upon progress in the modeling and computational evaluation of glass properties, a thermodynamic and semi‐empirical framework is established to delineate the accessible chemical space and to estimate key properties, including liquidus temperature, density, elastic modulus, and refractive index. This approach enables assessment of the attainable property realm, which is shown to far surpass that of oxide glasses, consistent with experimental trends observed in oxynitride systems. Prospective processing routes, functionalization strategies, and high‐performance applications are discussed in light of overcoming supply chain risks associated with materials in current use.

## Graphical abstract

Oxygen‐free all‐nitride glasses are revisited in light of new avenues of synthesis, raw material availability, and applications. A semi‐empirical analysis predicts mechanical performance achieving the level of engineering ceramics while maintaining visual transparency, and significant potential regarding optical properties into the infrared spectral range, overcoming supply chain limitations of critical chemical compounds.

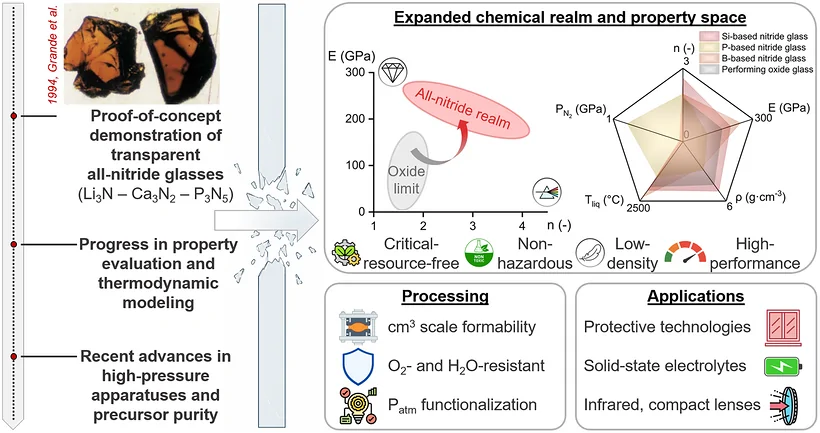

## 1 Introduction

Glasses, inorganic and non‐metallic, exhibit a unique combination of advantageous properties, including a theoretical strength on the order of 20 GPa, broad optical transmission windows, and exceptional chemical durability–all achievable through efficient and versatile processing techniques (e.g., liquid state shaping) [1, 2, 3]. These attributes underpin their indispensable role across numerous technological and everyday applications–a relevance highlighted during the International Year of Glass declared by the United Nations in 2022–largely governed by compositional fine‐tuning. Nevertheless, the deployment of glasses beyond mainstream applications–particularly in emerging contexts demanding high‐performance and resource‐efficient materials–remains constrained by the chemistry‐imposed limits on their accessible macroscopic properties [4], as schematically represented in Figure 1.

**FIGURE 1:**
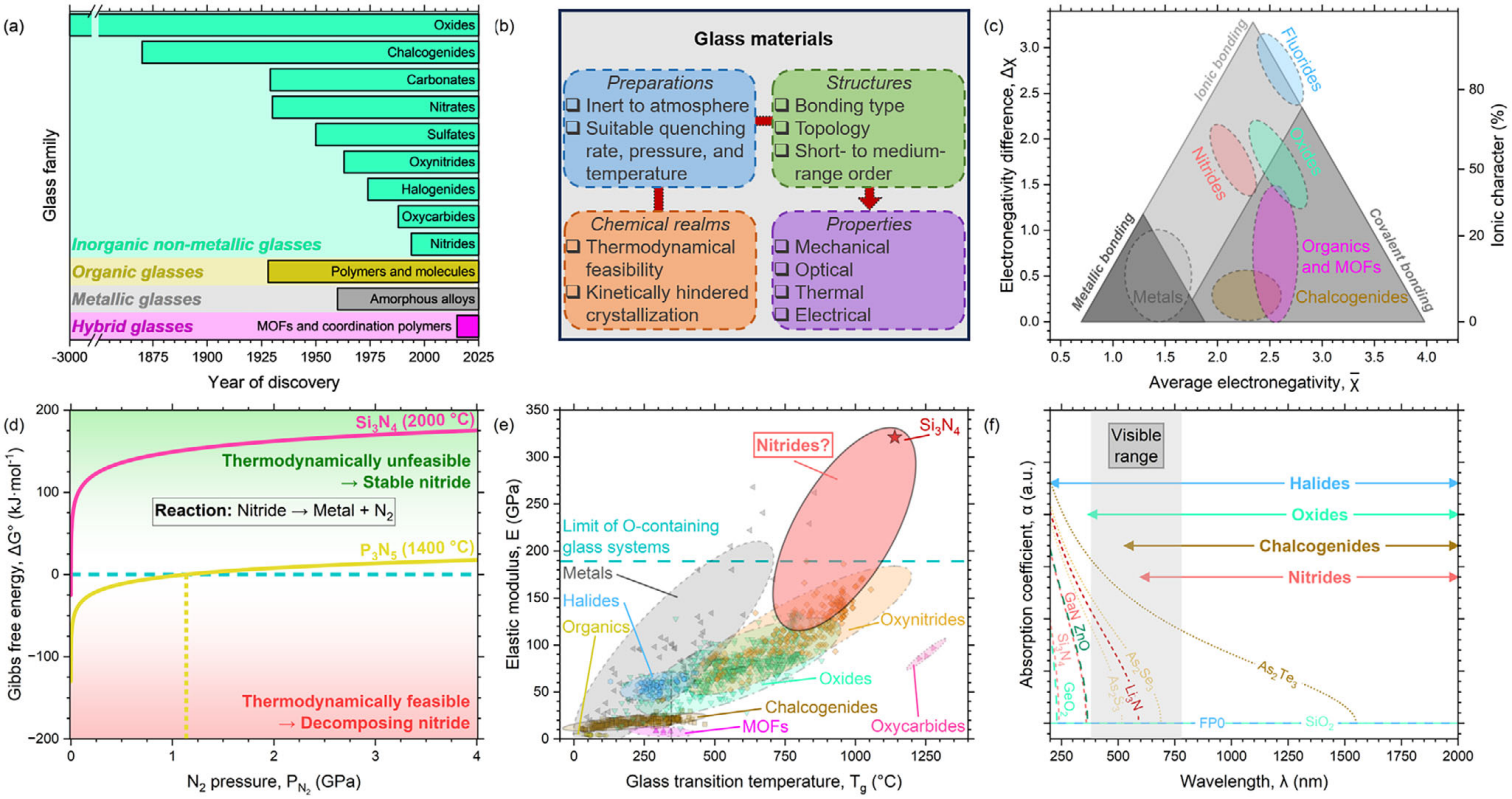
(a) Historical timeline illustrating the discovery of glass families and subfamilies [20, 23, 24, 25, 26, 27, 28, 29, 30, 31, 32, 33, 34]. MOF stands for metal‐organic framework. (b) Schematic representation of the conditions and means required for achieving glass materials exhibiting diverse structural features and macroscopic properties. (c) Representation of the chemical bonding of glass materials within the triangle of Arkel–Ketelaar. (d) N_2_‐pressure‐dependent Gibbs free energies of the decomposition reactions of the prototypical nitrides Si_3_N_4_ and P_3_N_5_ (representative of their conventional oxide glass‐formers counterparts, SiO_2_ and P_2_O_5_), for 1 mole N_2_, highlighting their domains of stability [35, 36]. (e) Elastic modulus and glass transition temperature across conventional glass families, be they organic, inorganic, or hybrid. Crystalline, sintered Si_3_N_4_ with low additive content (often oxide in nature, positioning at the grain boundaries within ceramics, thereby governing their high‐temperature creep behavior), for which the “transition temperature” was deduced from the temperature dependence of its elastic modulus, is indicated for comparison purposes [37]. The data were collected using the SciGlass database, and from relevant literature [13, 38, 39, 40, 41, 42, 43]. (f) Optical transmission windows of selected compounds [44, 45, 46, 47] derived using the Tauc plot equation for direct transition band gap [48] (neglecting contributions from indirect transition and absorption of hydroxyl groups or trace elements for clarity).

In fact, a consensus has emerged regarding the intrinsic physical boundaries of conventional methods–compositional and topological tailoring–which have stagnated over the past decades, and are unlikely to yield significant breakthroughs alone. Consequently, the pursuit of transparent yet robust materials remain a compelling challenge. This has spurred the exploration of alternative systems, notably of micro‐ and nano‐structured glass‐based materials (produced through controlled phase separation, crystallization, or composite routes), compatible with scalable processing techniques [5, 6, 7]. Although such approaches have yielded moderate gains (e.g., ∼20% increases in elastic moduli), they often entail processing complexity and partial loss of transparency [8]. This enduring “glass ceiling” emphasizes the need for fundamentally new strategies–for instance, departing from classical oxygen‐based systems towards new chemical territories.

Such strategies were employed as early as the 60s. Indeed, mixing distinct glass‐constituting anions has long served as a straightforward strategy in glass science to combine desirable attributes from different chemical systems without substantially complicating synthesis or reducing processing efficiency. Representative examples include oxyfluoride glasses, where fluorine F^−^ incorporation extends optical transparency into the deep ultraviolet while decreasing melting temperatures [9], and oxysulfide glasses, in which sulfide S^2−^ addition enhances electrical conductivity while retaining chemical durability [10]. Among such systems, however, the most profound improvements in mechanical performance arise from replacing 2‐fold oxygen O^2−^ with 3‐fold nitrogen N^3−^ within the glass network [11]. Irrespective of the cationic matrix, increasing nitrogen incorporation consistently leads to remarkable enhancements in mechanical properties (elastic moduli, hardness, fracture toughness)–yielding the largest values ever reported for inorganic, non‐metallic glasses, even six decades after their initial discovery [3]. Other mixed‐anion systems (such as oxycarbides, with 4‐fold C^4−^) or metallic glasses also provide mechanical reinforcement but fall short in key aspects [12, 13], either due to challenging processing routes (e.g., pyrolysis of preceramic polymers, necessity for high quenching rates) or compromised material properties (e.g., residual porosity, lack of optical transparency).

In spite of their advantageous properties and processing similarities to oxide systems, oxynitride glasses–although intensely studied from the 1970s onward–experienced a marked decline in research activity after the late 1990s [8]. The incorporation of nitrogen into phosphate and silicate matrices, typically achieved respectively via melt ammonolysis or the addition of nitride precursors to the batch (with melting conducted under protective N_2_ or Ar atmosphere) [14, 15], proved largely temperature‐dependent and was limited to a maximum O:N ratio of approximately 3:1 [3]. Efforts to increase nitrogen content necessitated increased temperatures to ensure melt homogeneity and sufficiently low viscosity for casting (or, even, to enable complete melting at all); however, such conditions inevitably approached the decomposition thresholds of nitride constituents under ambient pressure [16, 17]. While certain studies demonstrated suppression of these decompositions [18], notably through the application of high nitrogen pressures (on the order of 0.2 GPa, using a hot isostatic press) [19], the scarcity of suitable high‐pressure facilities offering sufficient quenching rates for glass formation restricted wider adoption. Consequently, the focus of research gradually shifted from controlling anionic to cationic compositions.

Nevertheless, a particularly noteworthy contribution came from Grande et al. (Angell's group, Nature, 1994) [20], who successfully synthesized about 20 small‐scale (∼50 mg) oxygen‐free nitride glass samples within the Li_3_N–Ca_3_N_2_–P_3_N_5_ chemical system. Enabled by the high‐pressure expertise of McMillan and Holloway [21, 22], this pioneering work demonstrated the feasibility of nitride glass formation. Although the study quantitatively reported only refractive indices, it established a proof of concept, demonstrating the feasibility of achieving fully oxygen‐free nitride glasses. The employed approach, based on trial and error, involved melting a nitride batch under sufficiently high pressures (up to 2.5 GPa, with a pure N_2_ atmosphere) to reach melting before decomposition using piston‐cylinder apparatuses. Indeed, at ambient pressure, few nitrides beside Li_3_N melt; the calculated evidence for P‐ and Si‐based nitride systems–reflecting conventional P‐ and Si‐based oxide glasses–, original to the present work and later described in Section 3.1, is presented in Figure 1d. These conditions, unattainable via conventional hot pressing, were at the time confined mainly to geoscience laboratories (given their capacity of replicating Earth's mantle conditions) which, together with the diminishing interest in N‐containing glass materials, limited continuity in the field.

In the present work, we revisit the original work and, drawing on predictive modeling and thermodynamical frameworks, as well as on advances in raw material purity and high‐pressure apparatus design over the past three decades–enabling larger sample volumes and improved operational accessibility–, provide new insights into these exotic glass materials. Particular emphasis is placed on processing‐dependent accessible compositional domains and on their physicomechanical (density, elastic modulus) and optical (refractive index, transmission window) properties. This approach highlights the potential to combine the hallmark transparency and processing versatility of glasses with the high‐performance attributes of technical ceramics, without dependence on resource‐intensive precursor materials. Finally, we outline prospective applications, targeted research pathways, and implications for the fundamentals of the glass transition.

## 2 Context and Implications of Early Works

### 2.1 All‐Nitride Glasses

The principal findings from the original investigations on all‐nitride glasses (comprising two publications and two patents [20, 21, 49, 50]) are summarized below, in the context of major data and knowledge accumulation in N‐containing glass systems that have occurred in the meantime (shown in Figure 2a). Approximately 70 nitride glass‐containing specimens (∼50 mg each; see photographs in Figure 2b,c and the curated dataset as extracted from the original study in Table S1) were synthesized under an N_2_ atmosphere at temperatures ranging from 650°C to 1500°C and pressures between 0.5 and 2.5 GPa. Dwell times varied from 20 to 240 min, followed by quenching at rates of approximately 100 K·s^−1^ (until 500°C), comparable to those employed in conventional oxide systems. In many cases, a preheating stage at lower temperatures and variable pressures preceded melting (30 min; 1000°C; 1–1.7 GPa). Representative furnace assemblies and experimental procedures are illustrated in Figure 2d,e. The investigated compositions were predominantly located within the Li_3_N–Ca_3_N_2_–P_3_N_5_ ternary system (shown in Figure 2f), where multicomponent compositions (≥ 3 nitrides), most likely those near deep eutectics, were observed to facilitate the formation of stable melts from which glasses may be derived, analogous to ZBLAN fluoride glass (only one binary composition, Li_4_PN_3_, formed a glass) [22]. Additional compositional variations included: (i) substitution of Ca_3_N_2_ with Mg_3_N_2_ or Ba_3_N_2_, (ii) substitution of Li_3_N with NaN_3_, (iii) incorporation of Si_3_N_4_, (iv) removal of alkaline earths (Li_4_PN_3_ binary system), (v) using commercial (oxygen‐containing) or lab‐synthesized P_3_N_5_, and (vi) use of precursors containing only P, N, and H or Cl. To this end, a double encapsulation technique was used [21, 51]. This configuration prevented both corrosion of the inner layer by the nitride melt and reactions between the outer layer and the ambient atmosphere (O_2_, H_2_O), achieved through the use of Ta (or Mo) and Pt metals, respectively. Samples were arbitrarily classified either as glass (< 10 vol.% of crystals), mixed glass/crystalline (∼50 vol.% of each phase), or crystalline (> 90 vol.% of crystals). It is worth mentioning that some compositions, while described in the patents as glassy, were presented for instance as decomposed in the original publication (e.g., pure P_3_N_5_).

**FIGURE 2:**
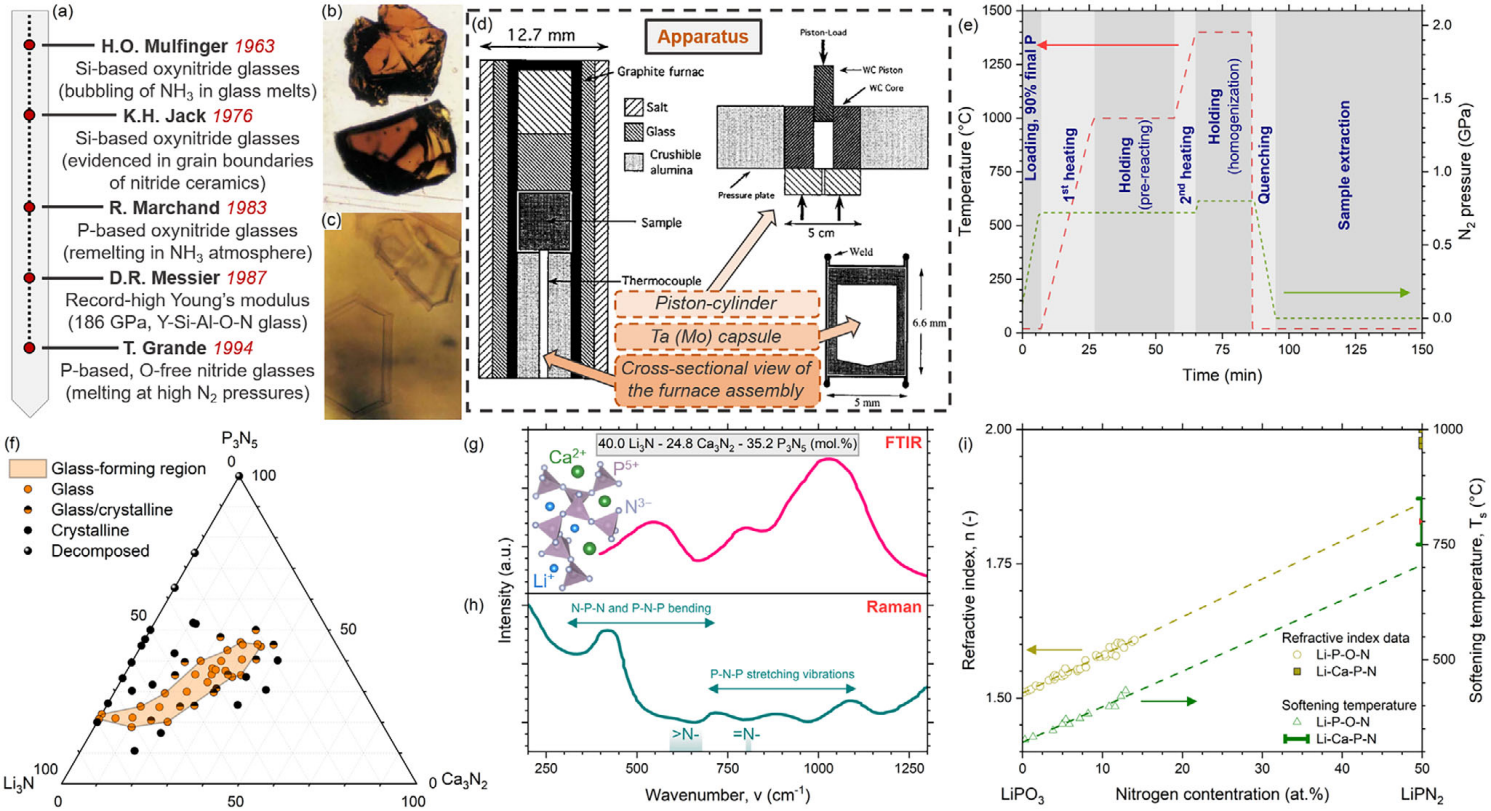
(a) Timeline depicting the major advances in N‐containing glass systems. Photographs of (b) nitride glasses (fragments of 0.1 to 1.0 mm in diameter), and (c) crystal‐containing nitride glass materials (with composition‐dependent sizes, from 10^0^ to 10^2^ µm) stemming either from incomplete melting, or crystallization during quenching (nitride glass‐ceramics). (d) Schematic illustrating the furnace assembly, the sample capsule (crucible), and the piston‐cylinder apparatus used for nitride glass synthesis. (e) Representation of the successive heating and pressurization steps for nitride glass synthesis. (f) Glass‐forming region in the Li_3_N–Ca_3_N_2_–P_3_N_5_ chemical system (mol.%). Glass, glass/crystalline, and crystalline describe materials with glass volume fractions of > 90, > 50, or < 10 vol.%. (g) FTIR, and (h) Raman spectra of the 40.0 Li_3_N–24.8 Ca_3_N_2_–35.2 P_3_N_5_ nitride glass. A structural model for the nitride glass structure is proposed in the present work in the inset. The emerging mode at ∼1200 cm^−1^ is a fluorescence band, attributed to potential Ta contamination. (i) Nitrogen concentration‐dependent refractive indices and softening temperatures in the Li–P–O–N chemical system in perspective to Li–Ca–P–N nitride glasses [20, 82]. Original data and photographs were reprinted or adapted from Refs. [20, 21] with permission.

Some insights into their structure were derived from Raman and Fourier‐transform infrared (FTIR) spectroscopies, both of which exhibited broad bands characteristic of amorphous systems as depicted in Figure 2g,h [20]. As direct assignment of vibrational modes by analogy with crystalline unary compounds (e.g., P_3_N_5_, Ca_3_N_2_, or Mg_3_N_2_ [52]) would be unsuitable, tentative structural interpretations were established between nitride and oxynitride glass systems, under the assumption that nitrogen occupies similar local environments in both cases and that the glass network is primarily composed of [PN_4_]^−7^ tetrahedral units, as observed in α‐P_3_N_5_ [53]. In P‐based oxynitride glasses, nitrogen incorporation induces but few modifications in FTIR spectra [54, 55, 56], mainly reflected by variations in the relative intensities of vibrational bands [57, 58]. Clearer trends are observed in Raman spectra, where two characteristic modes emerge and progressively increase in intensity with increasing nitrogen content at approximately 640 and 810 cm^−1^ [59, 60, 61, 62, 63]. These bands were attributed to triply coordinated (>N‐, N_t_) and doubly coordinated (= N‐, N_d_) nitrogen species, respectively. Comparable, although shiftedr features are observed in the Raman spectrum of the 40.0 Li_3_N–24.8 Ca_3_N_2_–35.2 P_3_N_5_ (mol.%) nitride glass. The presence of several other vibrational modes at frequencies similar to those reported for phosphate glasses, particularly in the high‐frequency region commonly associated with P–O–P and O–P–O linkages [59, 60, 63, 64], further suggests structural similarities between nitride and oxide glass networks. Variations in bond energies and bond angles [65] may account for the observed spectral shifts. It should nevertheless be noted that the dominant vibrational band centered near 400 cm^−1^ remains unassigned and may involve contributions from modifier cations, namely Ca^2+^ and Li^+^ [66]. In summary, these experimental observations suggest that nitride glasses, similarly to oxide glasses–and by analogy with chemically close crystalline counterparts–, are composed of a polymerized network of tetrahedra (here, [PN_4_]^−7^) interconnected through bridging nitrogen atoms, while modifier cations ensure charge compensation of non‐bridging nitrogen species.

Given the inherently exploratory nature of these early studies, only limited quantitative and qualitative characterization of the resulting materials was reported. The obtained samples were generally described as transparent, although occasional inhomogeneities were noted. Their visual appearance depended primarily on composition and crystallization rate, with reported colors spanning brown, red, orange, yellow, and green. This diversity reflects the broad optical behavior of nitride materials, which exhibit direct band gaps ranging from 0 to 7 eV [47]. Contamination from crucible materials, when observed, did not exceed 1 at.%. The amorphous nature of the samples was confirmed by X‐ray diffraction (XRD). As for the thermal properties of nitride glasses: viscous flow was observed between 750°C and 850°C, with their softening T_s_, glass transition T_g_, crystallization T_c_, and decomposition T_d_ temperatures (in N_2_, at ambient pressure) estimated to exceed 700–750°C, 700°C, 900°C, and 930°C, respectively. The crystallized phases were not reported. Mechanical properties included Mohs hardness values between 5.5 and 7.0 and Knoop hardness values above 280 HK. Notably, refractive indices ranging from 1.78 to above 2 were reported; quantitatively, this compares well with chemically comparable oxynitride glass systems, wherein a near‐linear trend along the join LiPO_3_–LiPN_2_ was noted (as shown in Figure 2i). This observation suggests that the compositional dependence of most physicomechanical properties of glasses on the nitrogen substitution rate N/(N+O) extends all the way to 100%. Despite these initial findings, the impact of these pioneering studies remained limited. This is attributable, in part, to the absence of quantitative characterization of key glass properties, including glass transition temperature, density, XRD patterns, and optical transmission spectra, but more decisively to the technical challenges involved at the time for synthesizing nitride glasses, combined with a waning interest in oxynitride glasses. Consequently, the echo amongst peers remained limited (∼30 citations since), predominantly consisting of qualitative references, without any attempts at theoretical or experimental replication or continuation by either Angell's group or other laboratories [51, 60, 67, 68, 69, 70, 71, 72, 73, 74, 75, 76, 77, 78, 79, 80, 81].

### 2.2 Extrapolating Oxynitride Glasses

Oxynitride glasses are glasses wherein 3‐fold nitrogen atoms substitute 2‐fold oxygen atoms [8, 83]. First reported in the 60s [28], and subsequently identified in the 70s within intergranular glassy phases of silicon nitride ceramics sintered with oxide sintering additives [84], several synthesis routes have since been established. These include ammonolysis of glass melts and gels [85, 86], as well as the incorporation of nitrides, metals, and hydrides into the initial batch [11, 87, 88]. Most of the periodic table has been explored [89, 90], and systematic variation of both cationic and anionic compositions now enable a comprehensive representation of the accessible property space, as summarized in Figure 3a–i.

**FIGURE 3:**
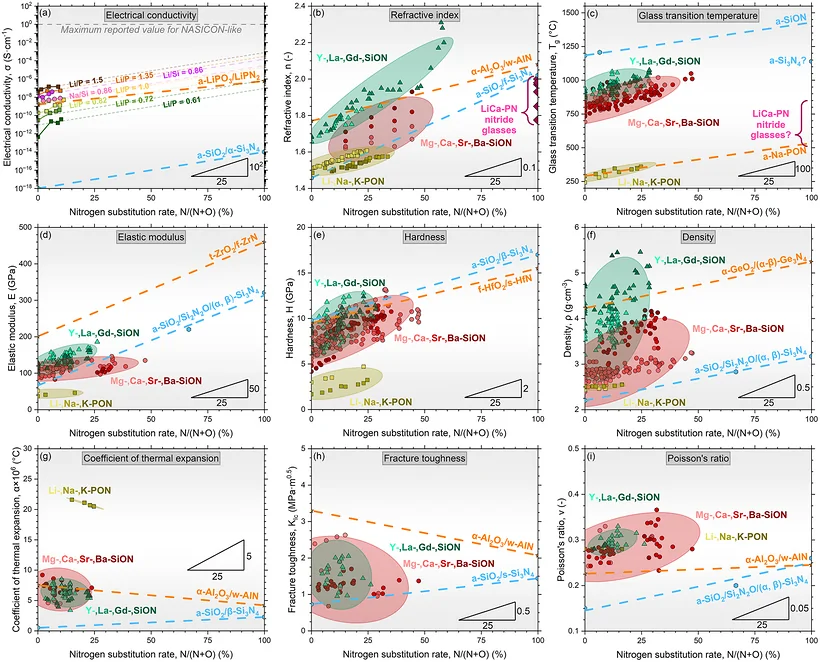
Dependence of selected macroscopic properties on nitrogen substitution in various oxynitride glass matrices: (a) electrical conductivity; (b) refractive index; (c) glass transition temperature; (d) elastic modulus; (e) hardness; (f) density; (g) coefficient of thermal expansion; (h) fracture toughness; (i) Poisson's ratio. For enhanced clarity, systems involving three or more anions (e.g., oxyfluoronitrides, oxycarbonitrides, or oxysulfonitrides) are not included [10, 91, 92, 93]. Corresponding trends for simple oxides and nitrides with identical cations–albeit, in some cases, with differing oxidation states–are also indicated. The reported values for crystalline materials were obtained from samples of diverse morphologies and microstructures (e.g., fully dense or porous specimens, processed with or without sintering additives, occasionally containing multiple polymorphs, as well as thin films and single crystals) and under varying measurement conditions (e.g., wavelength for refractive index, loading conditions for hardness, orientation relative to crystallographic axes for the thermal expansion coefficient). Consequently, these data are included primarily for illustrative and comparative purposes. The notations f‐ and s‐ respectively denote thin films and single crystals and, when multiple polymorphs can exist (or coexist), the following designations are used: a‐ (glassy), w‐ (wurtzite), t‐ (tetragonal), h‐ (hexagonal), α‐ (alpha), and β‐ (beta). The data were collected from relevant literature [3, 8, 20, 21, 37, 38, 49, 50, 55, 56, 60, 63, 67, 82, 83, 87, 90, 93, 94, 95, 96, 97, 98, 99, 100, 101, 102, 103, 104, 105, 106, 107, 108, 109, 110, 111, 112, 113, 114, 115, 116, 117, 118, 119, 120, 121, 122, 123, 124, 125, 126, 127, 128, 129, 130, 131, 132, 133, 134, 135, 136, 137, 138, 139, 140, 141, 142, 143, 144, 145, 146, 147, 148, 149, 150, 151, 152, 153, 154, 155, 156, 157, 158, 159, 160, 161, 162, 163, 164, 165, 166, 167, 168, 169, 170, 171, 172, 173, 174, 175, 176, 177, 178, 179, 180, 181, 182, 183, 184, 185, 186, 187, 188, 189, 190, 191, 192, 193, 194, 195, 196, 197, 198, 199, 200, 201, 202, 203, 204, 205, 206, 207, 208, 209, 210, 211, 212, 213, 214, 215, 216].

A general trend emerges: for a fixed cationic composition, and regardless of whether the system is Si‐ or P‐based, an increase in the nitrogen substitution ratio, N/(N+O), results in a strong and approximately linear increase in most macroscopic properties. These include electrical conductivity (on a logarithmic scale), refractive index, glass transition temperature, elastic modulus, hardness, and density (see Figure 3a–f). Similar tendencies are observed for the coefficient of thermal expansion, fracture toughness, and Poisson's ratio, albeit with greater scatter (as shown in Figure 3g–i). Consequently, further enhancement of the performance of inorganic, non‐metallic glasses via nitrogen incorporation is constrained by the attainable substitution levels–typically one nitrogen atom per three oxygen atoms–and, therefore, by the thermally induced decomposition of nitrides at ambient pressure. Extrapolation of these linear relationships to complete substitution of oxygen by nitrogen (supported by trends observed in all‐nitride glasses for refractive indices and softening temperatures [20]) suggests, for instance, improvements such as an increase in electrical conductivity by up to four orders of magnitude and an enhancement of the elastic modulus by a factor of 2–3. This therefore offers an original pathway to surpass the long‐standing performance ceilings of oxide and mixed‐anion inorganic glasses, towards the development of processable, high‐performance glass materials–whose prospective macroscopic properties are further discussed in Section 3.

## 3 Results and Discussion

### 3.1 Stability Domains of Nitrides

At ambient pressure, Ellingham diagrams–representing the temperature dependence of the Gibbs free energy change (ΔG°) associated with chemical reactions–can be readily computed from thermochemical data, including standard molar entropies and formation energies of the relevant compounds. Such diagrams provide reliable estimates of temperature‐dependent phase stability, not only for simple systems (e.g., CuO/Cu_2_O undergoing thermally activated decomposition), but also for more complex, multicomponent systems such as oxynitride glass‐forming melts, wherein competing processes including decomposition, reduction, and nitriding can occur [89].

However, the synthesis of nitride glasses requires conditions beyond ambient pressure, rendering Ellingham diagrams insufficient for assessing the thermal stability of nitrides under such conditions. In pressure–temperature (P–T) diagrams, both enthalpic (including the pressure‐volume term) and entropic contributions to the Gibbs free energy must be considered. While methodologies such as CALPHAD (Calculation of Phase Diagrams) enable the treatment of volumetric effects at elevated pressures, their implementation requires compound‐specific fitting of equation‐of‐state parameters [217]. This requirement introduces significant challenges, compounded by the experimental difficulty associated with synthesizing some nitrides, but also because some thermochemical properties–such as melting enthalpies–, though of importance for calculating P–T diagrams, were never determined experimentally, primarily because few nitrides melt at near‐ambient pressures.

In the present study, considering pressures up to approximately 4 GPa (the upper limit of conventional piston‐cylinder apparatuses), pressure‐induced increases in melting temperature are limited to approximately 50–100 K for simple oxides [218, 219], oxide mixtures [220], and simple nitrides [221, 222]. Consequently, the volume contribution to the Gibbs free energy is assumed to be negligible, and the calculated P–T diagrams are interpreted herein as approximate indicators of nitride stability, with acceptable uncertainties of this magnitude. P–T diagrams were straightforwardly computed using CALPHAD modeling implemented in the well‐established FactSage software suite (FactPS and SpMCBN databases) [223, 224, 225, 226], considering elements up to the early actinides (up to Cf). The results are presented in Figure 4a–f with respect to the accessible temperatures (up to 2300°C) and pressures for commercially available piston‐cylinder apparatuses.

**FIGURE 4:**
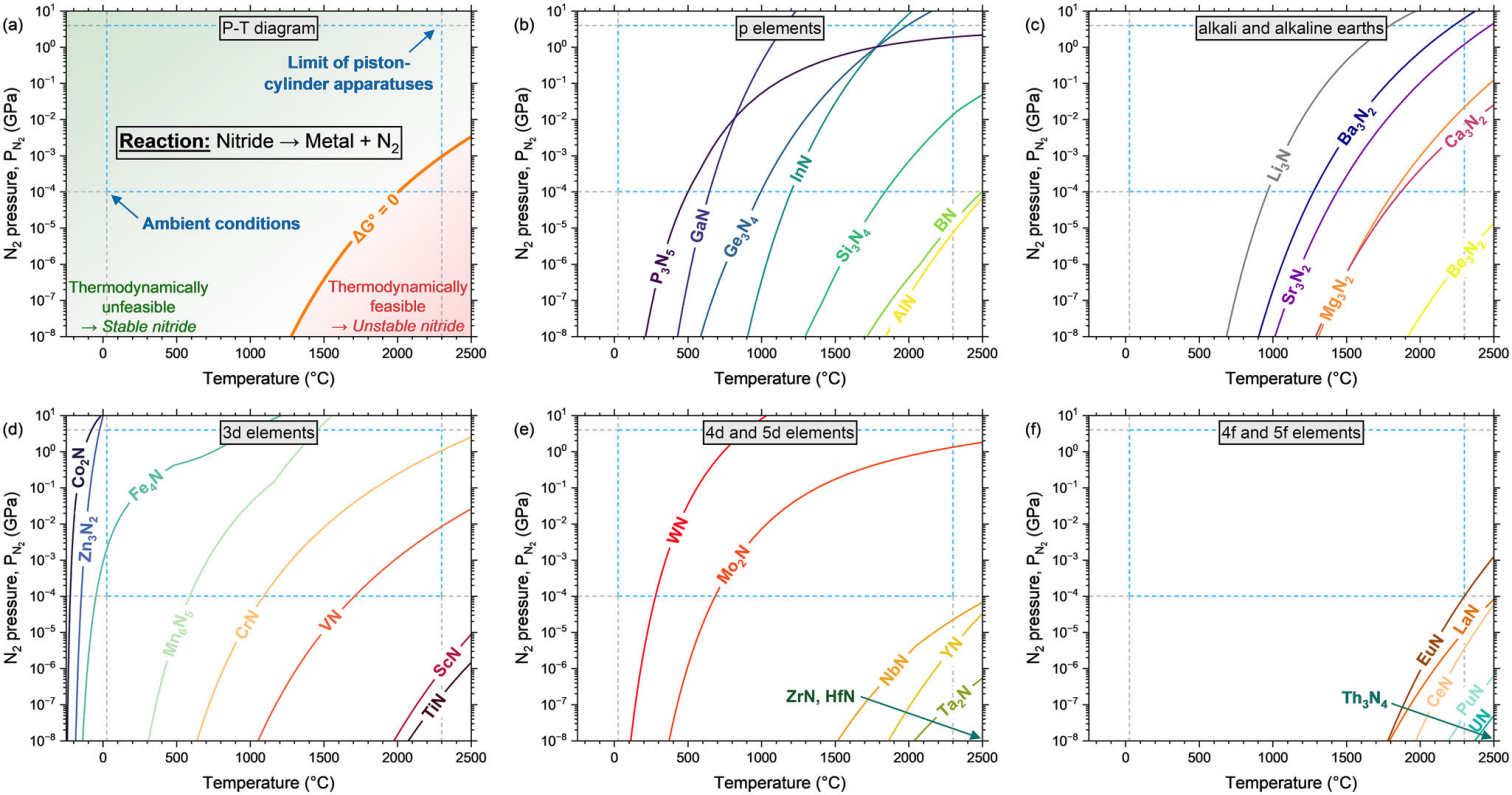
(a) Scheme highlighting the stability regions of a fictional nitride (be it solid or liquid) in a pressure‐temperature (P–T) diagram, considering the range of accessible pressures and temperatures with conventional piston‐cylinder apparatuses, albeit limits–as well as associated capsule volumes and corresponding sample dimensions–are contingent upon the specific equipment provider [229, 230, 231]. Stability regions of various nitrides in the P–T diagram, calculated using all available phase transitions of the involved metallic, gaseous, and nitride compounds; when multiple nitrides may co‐exist (e.g., TaN and Ta_2_N), only the nitride bordering the metal + N_2_ region is shown: (b) p elements, (c) alkali and alkaline earths, (d) 3d elements, (e) 4d and 5d elements, and (f) 4f and 5f elements. Highly stable nitrides lying outside the considered window (ZrN, HfN, and Th_3_N_4_) are presented as such. Nitrides for which no thermochemical data were available, or which show no stability in the considered processing window, are not presented.

The calculated results exhibit satisfactory agreement with experimental observations. For instance, pressures on the order of 1 GPa suppress the decomposition of P_3_N_5_ in all‐nitride melts at 1400 °C, thereby enabling melting and subsequent quenching into nitride glasses [20, 21], whereas at 1900°C, pressures of 0.2 GPa (or, rather, any pressure beyond 1 MPa, i.e., only ten times the atmospheric pressure) inhibit the decomposition of Si_3_N_4_ in oxynitride melts (see Figure 4b) [19]. Other examples demonstrating good agreement include Mo_2_N (complete decomposition experimentally observed at 880°C [227]; 793°C calculated) and GaN (onset of decomposition at 710°C [228]; 646°C calculated); it should be noted that slight deviations are common, as this thermodynamic approach does not account for decomposition kinetics. Despite incomplete thermochemical datasets for certain nitrides, it appears that the accessible compositional space for nitride glasses is more restricted than that for oxide glasses. Several 3d transition metal nitrides (e.g., Ni_3_N, Co_2_N) are unstable under the investigated conditions, and only one alkali nitride (Li_3_N) is stable (as shown in Figure 4c,d). Nevertheless, considering reasonable melting temperatures of at least 1000 °C to melt a multicomponent nitride melt, it is estimated that approximately 30 nitrides may be incorporated into nitride glass systems (as detailed in Section 3.4), compared to roughly 60 oxides in oxide glass systems [38].

### 3.2 Prediction of Macroscopic Properties

With the compositional domain of thermodynamically accessible nitride glass systems now delineated, attention can be directed towards estimating their mechanical performance in order to assess material handling and the preservation of structural integrity during service. In glass science, the model proposed by Makishima and Mackenzie in the 70s remains one of the most renowned and widely employed approaches for this purpose [232, 233]. Despite recognized limitations–for instance in the case of borate glasses [3, 234, 235]–it provides a reasonable estimation of the elastic modulus E for oxide‐based systems, and beyond. Moreover, the model is computationally straightforward, requiring only the nominal composition, the packing density (C_g_), and tabulated thermochemical data of constituent compounds:

(1) $$E=2\;\times \;C_{g}\times \;G$$

where G denotes the volume dissociation energy. The packing density, which reflects the atomic packing efficiency and thus the structural organization of the glass network, is determined from the molar volume of the glass (V_m_), itself computed from the ionic radii (r_i_) of the constituent ions i:

(2) $$V_{m}=\frac{\sum_{i}f_{i}M_{i}}{\rho }\;$$

(3) $$C_{g}=\frac{V_{t}}{V_{m}}=\frac{N\sum_{i}\frac{4}{3}\pi f_{i}{r_{i}}^{3}}{V_{m}}\;$$

here, f_i_​ and M_i_​ denote the atomic fraction and molar mass of the i^th^ ion, respectively, ρ is the glass density, and N is Avogadro's number. The term V_t_ corresponds to the theoretical volume occupied by ions, considering a hard sphere model.

The CaO–Al_2_O_3_–SiO_2_ ternary system is considered here as a representative case study, as it encompasses a broad glass‐forming domain that is well suited for illustrating the applicability of the proposed approach. Within this compositional space, approximately 100 elastic modulus values were reported [38]. The calculated elastic moduli (E_calculated_) show good agreement with the experimental values (E_measured_) across the entire compositional range, albeit with a certain offset of ∼10 GPa (see Figure 5a–c). This discrepancy can be attributed to the composition‐dependent formation of [AlO_5_] and [AlO_6_] polyhedra at the expense of [AlO_4_] tetrahedra [236], which are not accounted for in the Makishima and Mackenzie model. Most importantly, it can be seen that the elastic modulus is primarily governed by the volume dissociation energy, as both exhibit identical compositional trends, whereas the packing density remains relatively constant, varying only slightly around 51% ± 2%, as shown in Figure 5d–f.

**FIGURE 5:**
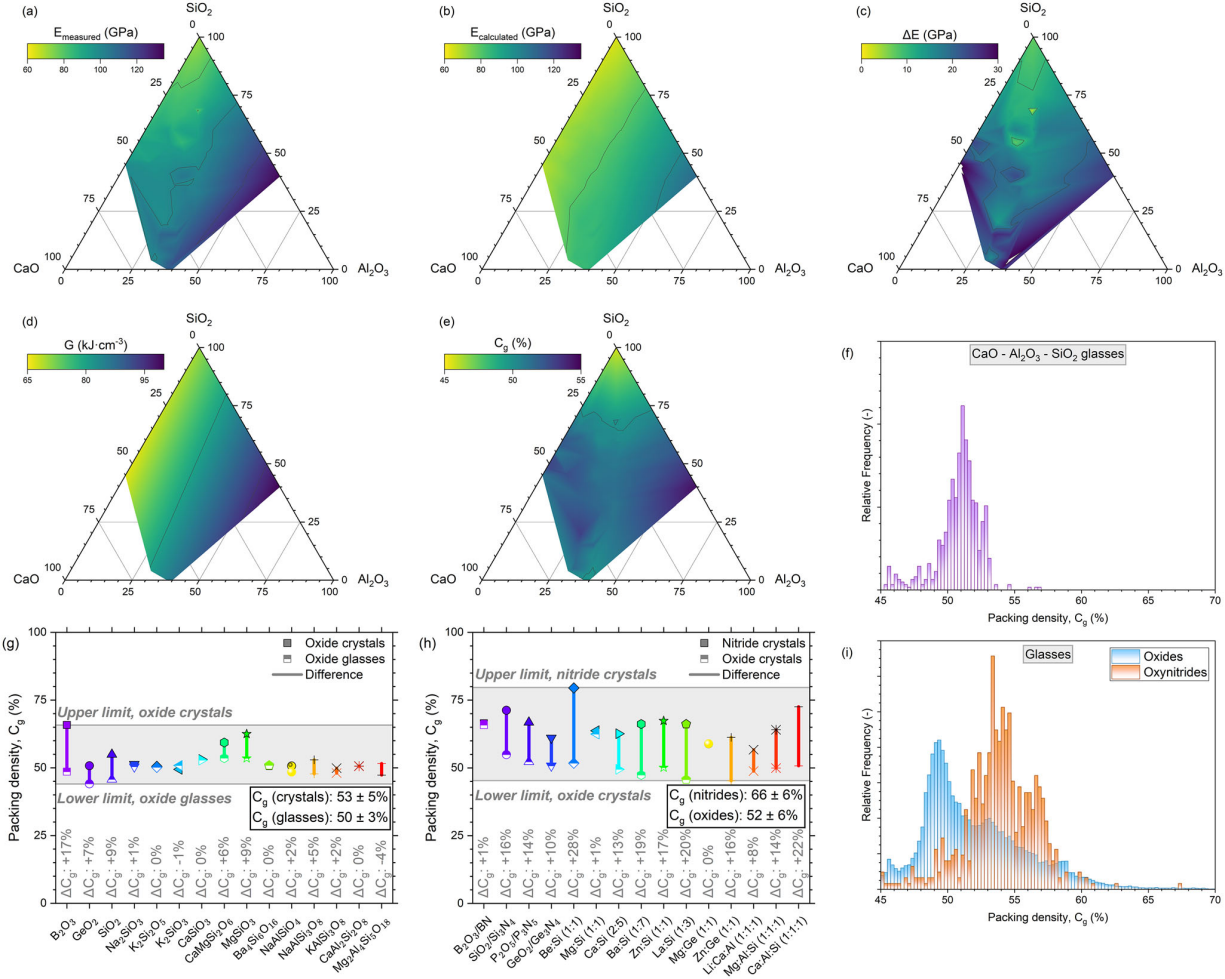
Compositional dependence in the CaO–Al_2_O_3_–SiO_2_ ternary diagram of (a) the measured elastic moduli (E_measured_), (b) the elastic moduli calculated (E_calculated_) using the model of Makishima and Mackenzie and experimental density values [232], (c) the difference between measured and calculated elastic moduli (ΔE), (d) the volume dissociation energy (G), and (e) the packing density (C_g_). (f) Relative frequency of packing densities in the CaO–Al_2_O_3_–SiO_2_ ternary diagram. Comparison of the packing densities of (g) oxide crystals and oxide glasses, and (h) nitride crystals and oxide crystals. (i) Relative frequency of packing densities in oxide and oxynitride glass systems. Data (densities, ionic radii, thermochemical properties, and elastic moduli) were obtained from the SciGlass database and relevant literature [35, 36, 38, 90, 238, 239].

Nevertheless, obtaining reliable estimates of both the magnitude and compositional dependence of elastic moduli in nitride glasses requires consideration of both the volume dissociation energy and the packing density. In fact, the original model was extended in the 80s by Rocherullé et al. to account for nitrogen‐containing systems through incorporation of the ratio of formation free energies of monoatomic nitrogen and oxygen (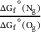
= 1.98) in the evaluation of the volume dissociation energy [167]. This modification enabled satisfactory agreement between calculated and experimental elastic modulus values for oxynitride glasses. However, the approach remains inherently semi‐empirical, as it requires prior knowledge of the glass density–and thus of the packing density–for its implementation. While reliable density estimates can be obtained for conventional oxide systems from extensive databases such as SciGlass (> 400 000 individual entries) [38, 43], this is not the case for nitride glasses, for which no experimental density data exist.

To address this limitation, packing density values were compiled from systems with identical cationic compositions and comparable chemical bonding, including oxide crystals versus oxide glasses (Figure 5g), as well as nitride crystals versus oxide crystals (Figure 5h). Overall, oxide crystals are more efficiently packed than oxide glasses by 3% on average (though a few, minor exceptions exist), whereas nitride crystals are drastically more efficiently packed than oxide crystals, by 14% on average. This observation is consistent with trends in conventional oxynitride glasses, wherein partial substitution of oxygen by nitrogen up to one in four results in an increase in packing density of up to approximately5% (see Figure 5i). Accordingly, as a first‐order approximation, the packing densities of nitride glasses are assumed to be approximately 3% lower than those of the corresponding crystalline phases of the constituent glass formers (e.g., considering a packing density of 71% for Si_3_N_4_, this yields an estimated packing density of 71–3 = 68% for Si‐based nitride glasses), yielding an average value of about 63%. Note that this also allows estimating the density of such systems. When applied to representative systems such as glassy SiO_2_ and crystalline Si_3_N_4_, the resulting estimates (62 and 257 GPa) are systematically underestimated but remain in reasonable agreement with experimental values (70 and 321 GPa) [3, 37]. This correspondence suggests that even a simplified energetic framework can provide reliable estimates of the elastic moduli of nitride glasses. A detailed calculation sheet (Table S2a,b) is provided in the Supporting Information for conducting calculations of the packing density and elastic modulus for multicomponent oxide and nitride systems.

It is further noted that additional mechanical properties, including hardness, Poisson's ratio, and viscosity, are anticipated to increase relative to oxide‐based systems. In particular, fracture toughness (K_Ic_) values reported for oxide glasses typically fall within the range of 0.4 to 0.8 MPa·m^0.5^, as determined by self‐consistent methods [89, 237]. On the basis of Rouxel's model, and assuming comparable chemical spaces (i.e., accessible cationic compositions) for oxide and nitride systems, nitride glasses are expected to exhibit fracture toughness values approximately 50%–100% larger than those of their oxide counterparts. This trend is consistent with comparative data for glassy SiO_2_ and Si_3_N_4_ single crystals, which show values of 0.72 and 1.44 MPa·m^0.5^, respectively [8, 208].

Beyond mechanical properties, optical properties–particularly the refractive index–constitute another key class of parameters for glass materials, especially in the context of photonic applications. Although no simple semi‐empirical model exists that allows the refractive index of glasses to be estimated solely from composition, analogous to the Makishima‐Mackenzie model for elastic moduli, an alternative approach is proposed herein. A central element of this framework is the extension of the well‐established optical basicity concept–reflecting the electron‐donating ability of a matrix–, which was initially developed for oxide systems [240], then applied to fluoride systems [241], and more recently extended to (non‐glassy) nitride systems (see Ref. [239]). In this context, the optical basicity of simple nitrides (Λ_N_) is derived from that of the corresponding oxides (Λ_O_) according to:

(4) $$\Lambda_{N}=\frac{3}{2}\;\Lambda_{O}$$

Following Duffy's definition of optical basicity [240], the theoretical optical basicity (Λ_th_) of multicomponent nitride systems can be determined from the stoichiometry (i.e., the proportion of nitrogen atoms). For instance, a hypothetical equimolar Li_3_N–Ca_3_N_2_–P_3_N_5_ glass yields:

(5) $$\Lambda_{\mathrm{th}}=\frac{1}{8}\;\Lambda (Li_{3}N)+\frac{2}{8}\Lambda (Ca_{3}N_{2})\frac{5}{8}\Lambda (P_{3}N_{5})=0.91$$

From this value, the nitrogen polarizability (*α*_N_) can be deduced as:

(6) $$\alpha_{N}=2.56\Lambda_{\mathrm{th}}+0.37$$

The molar polarizability (α_m_) is then obtained by incorporating the polarizability (α_Ci_) of each cation i:

(7) $$\alpha_{m}=N_{N}\;\alpha_{N}+\sum_{i}{N_{C}}_{i}{\alpha_{C}}_{i}$$

where N_N_ and N_Ci_ denote the numbers of nitrogen ions and cations i, respectively. The molar refractivity (R_m_) follows as:

(8) $$R_{m}=2.52\alpha_{m}$$

The molar refractivity is connected to both the molar volume and the refractive index (n) through the Lorentz‐Lorenz equation [242]:

(9) $$R_{m}=V_{m}\;\frac{n^{2}\;-\;1}{n^{2}+2}$$

Finally, combining this relation with the previously defined packing density (and thus molar volume), the refractive index can be expressed as:

(10) $$n=\sqrt{\frac{\frac{2R_{m}C_{g}}{V_{t}}+1}{1\;-\;\frac{R_{m}C_{g}}{V_{t}}}}\;$$

A detailed calculation sheet (Table S3a,b) is provided in the Supporting Information to enable the computation of theoretical optical basicity and refractive index for any multicomponent oxide and nitride system.

For validation purposes, the proposed approach is first applied to the previously investigated CaO–Al_2_O_3_–SiO_2_ ternary system. The measured (n_measured_) and calculated (n_calculated_) values of the refractive index are respectively presented in Figure 6a and Figure 6b, together with their difference (Δn) and the corresponding theoretical optical basicity in Figure 6c,d, respectively. Overall, both measured and calculated refractive indices are observed to scale directly with optical basicity. Although deviations are present, reaching up to ± 0.065 in rare instances (i.e., up to ∼4% relative variation), these differences remain limited, indicating that the proposed, simple approach satisfactorily reproduces compositional trends in glass systems. Furthermore, application to simple nitride systems, as well as to the limited set of values reported in early studies on nitride glasses, yields excellent agreement between calculated and experimental refractive indices, as summarized in Table 1.

**FIGURE 6:**
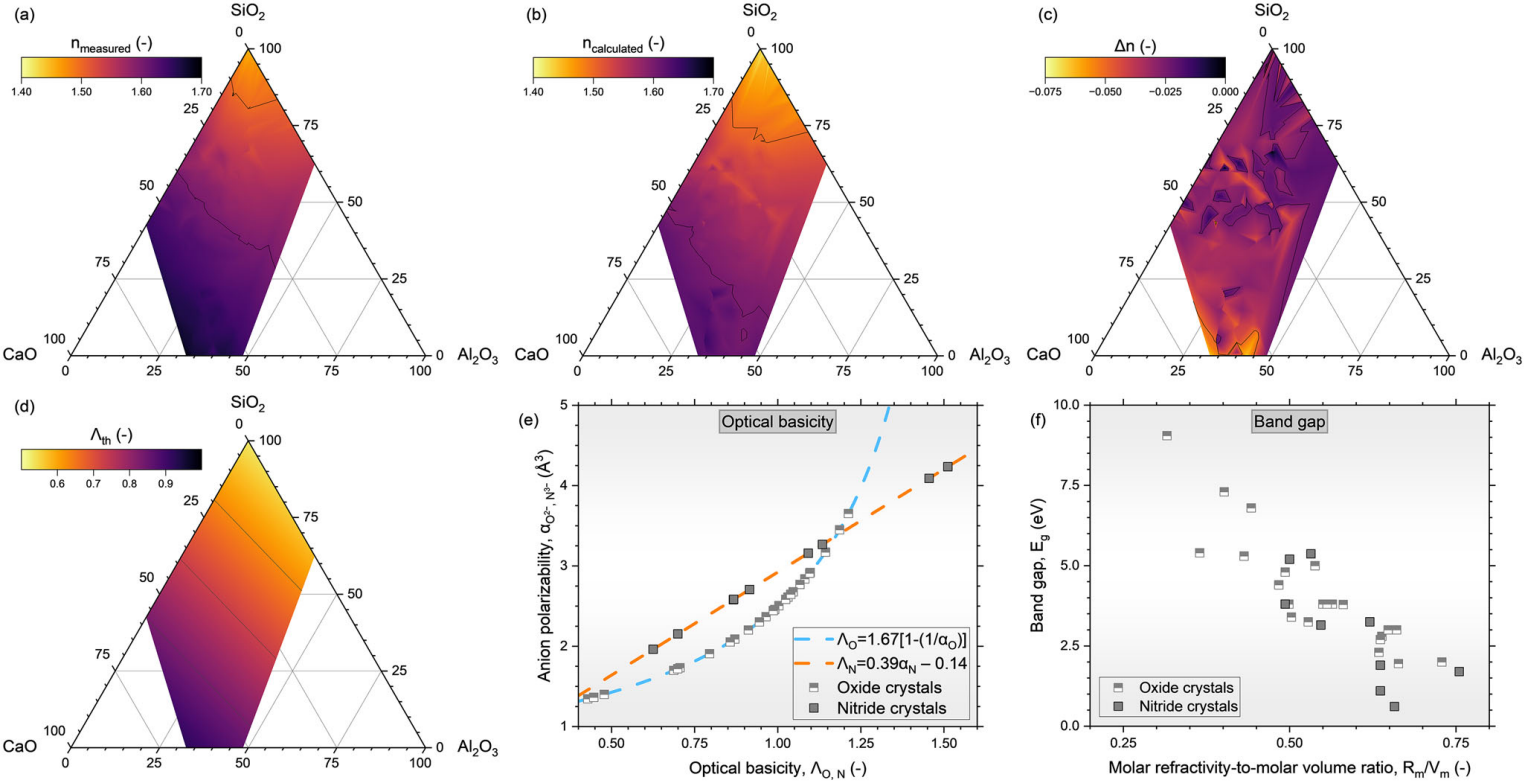
Compositional dependence in the CaO–Al_2_O_3_–SiO_2_ ternary diagram of (a) the measured refractive indices (n_measured_), (b) the calculated refractive indices (n_calculated_) using theoretical optical basicity values and experimental density values, (c) the difference between measured and calculated refractive indices (Δn), and (d) the theoretical optical basicity (Λ_th_). (e) Dependence of the anion polarizability on the optical basicity for oxide and nitride systems. (f) Molar refractivity‐to‐molar volume ratio of simple oxide and nitride crystals. Data (ionic radii, cationic polarizabilities, refractive indices, equations for calculating optical basicity values from anionic polarizabilities, and band gaps) were obtained from the SciGlass database and relevant literature [38, 44, 47, 238, 239, 240, 244].

**TABLE 1:** Comparison of measured and calculated refractive indices using the proposed approach for representative glassy and crystalline nitride systems. Measured values for crystalline nitrides are taken from Refs. [192, 194, 243]. Refractive indices were determined using the oil immersion method for nitride glasses, and at a wavelength of 546 nm for nitride crystals [20].

Nitride systems	Compounds	n_measured_ (‐)	n_calculated_ (‐)	Difference (‐)	Relative error (%)
Glasses	56.2 Li_3_N – 14.4 Ca_3_N_2_ – 29.4 P_3_N_5_	1.98	1.95	0.03	1.5
	71.1 Li_3_N – 10.5 Ca_3_N_2_ – 28.4 P_3_N_5_	1.97	2.01	−0.04	2.0
	77.4 Li_3_N – 22.6 P_3_N_5_	1.85	1.91	−0.06	3.2
Crystals	α‐Si_3_N_4_	2.02	2.02	0.00	0.0
	w‐AlN	2.08	2.17	0.09	4.3
	h‐BN (out‐plane index, ⟂)	1.88	1.81	−0.07	3.7

Finally, although transmission spectra were not reported in the original nitride glass studies, indirect evidence based on their coloration (ranging from red to green [20]) and the generally smaller band gaps of nitrides [47] compared to oxides [44] (as shown in Figure 6f) suggests that both the optical transmission window (in the visible range) and the band gap of nitride glasses are smaller relative to conventional oxide glasses. Coupled with their demonstrated transparency, these characteristics thereby motivate investigation into their potential for electronic and optoelectronic applications, and warrant further studies towards developing transparent and colorless all‐nitride glasses.

### 3.3 Representative Nitride Glass Systems

The accessible property space of nitride glasses is inherently constrained by the extent of their glass‐forming domains and the nature of their structural configurations. Evidence from the seminal study on nitride glasses suggests that their structures more closely resemble those of oxide glasses–both characterized by a glass network backbone involving tetrahedra–than those of fluoride glasses, which typically exhibit higher cation coordination numbers [245]. This distinction is plausibly attributable to the comparatively lower covalent character of bonding in fluoride systems. Supporting this interpretation are (i) the partially overlapping glass‐forming regions observed for oxide and nitride compositions within the M^+^–P^5+^–M^2+^ ternary system (where M^+^ and M^2+^ denote alkali and alkaline earth cations, respectively), as shown in Figure 7e and (ii) the contrasting limited solubility of P^5+^ species in fluoride glasses (< 3 at.%) [38].

**FIGURE 7:**
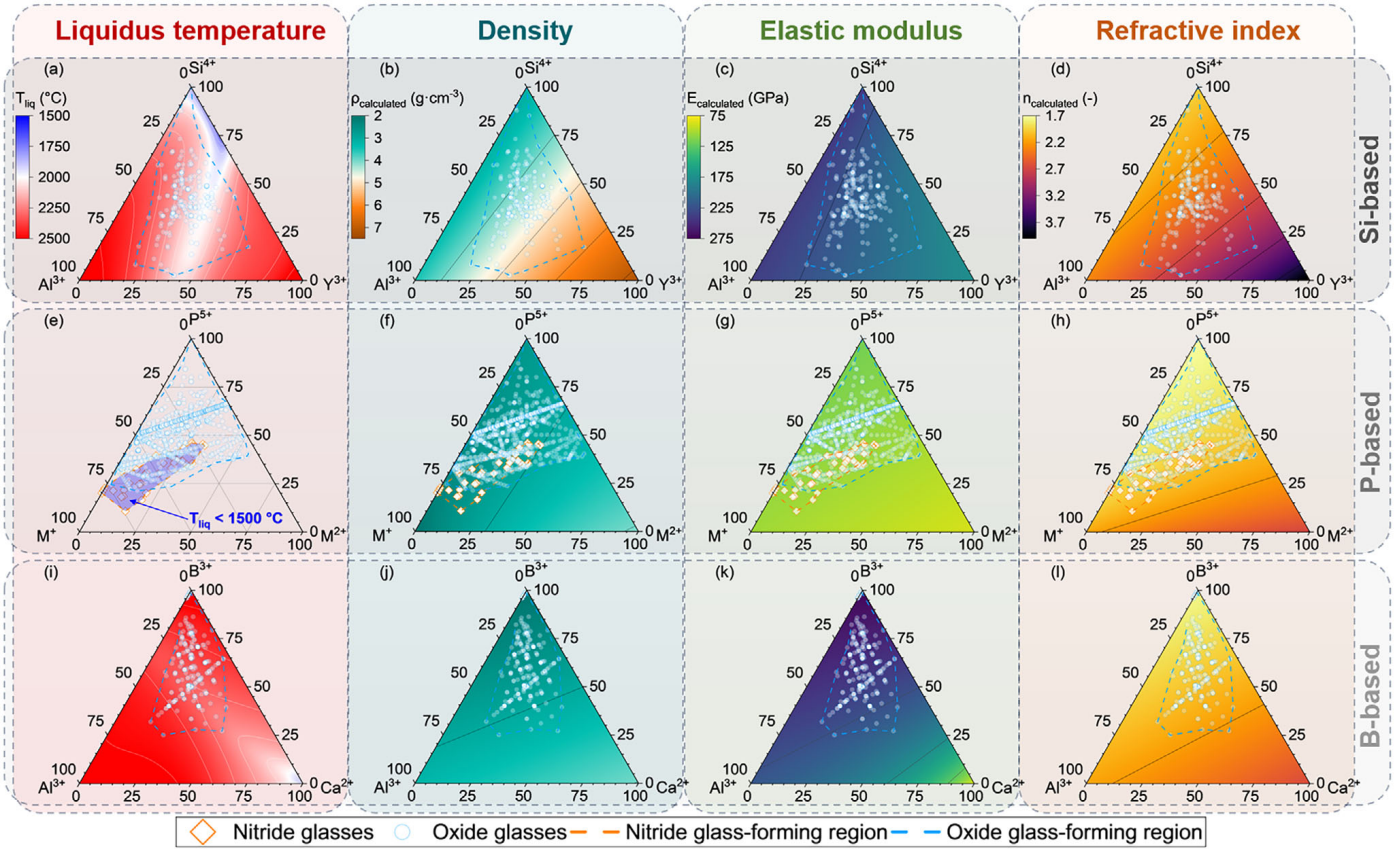
Computed properties in the Y^3+^–Si^4+^–Al^3+^ nitride glass chemical system: (a) liquidus temperature, (b) density, (c) elastic modulus, and (d) refractive index. Computed properties in the Li^+^–P^5+^–Ca^2+^ nitride glass chemical system: (e) liquidus temperature, (f) density, (g) elastic modulus, and (h) refractive index. Computed properties in the Ca^2+^–B^3+^–Al^3+^ nitride glass chemical system: (i) liquidus temperature, (j) density, (k) elastic modulus, and (l) refractive index. Individual data points corresponding to oxide glasses (and nitride glasses, where available) are indicated, with contours delineating the glass‐forming regions [38]. As few oxide glass compositions were reported in the Li^+^–P^5+^–Ca^2+^ chemical system, they are instead projected from the broader M^+^–P^5+^–M^2+^ oxide glass system (where M^+^ is an alkali, from Li to Cs, and M^2+^ is an alkaline earth, from Be to Ba). The Li_3_N and P_3_N_5_ compounds are not included in the SpMCBN database of the FactSage software suite used for liquidus temperature calculations of multicomponent nitride mixtures; consequently, the liquidus temperatures are represented qualitatively based on prior studies [20].

On this basis, three representative nitride glass systems are selected by analogy to well‐established oxide glass families: (i) a Si‐based system (Y^3+^–Si^4+^–Al^3+^), noted for its potential for superior mechanical performance [8, 173]; (ii) a P‐based system (Li^+^–P^5+^–Ca^2+^), derived from the original nitride glass investigations and relatively underexplored in terms of macroscopic properties [20]; and (iii) a B‐based system (Ca^2+^–B^3+^–Al^3+^), notable for its small density [38]. Liquidus temperatures (T_liq_, the temperature at which a composition is completely liquid, free from any solid phase) were calculated using CALPHAD modeling implemented in the FactSage software suite [223, 224, 225, 226], while density, refractive index, and elastic modulus were determined following the methodology outlined in Section 3.2. The resulting property maps are presented in Figure 7a–l, expressed in cationic percent (cat.%) to enable comparison with oxide systems.

The Si‐based system (see Figure 7a–d) exhibits the largest predicted refractive indices (up to ∼3.2), along with large elastic moduli (up to ∼240 GPa) and densities (up to ∼6 g·cm^−3^), and encompasses a substantial compositional domain of liquidus temperatures accessible using conventional piston‐cylinder apparatuses (< 2300°C). The P‐based system represents a lightweight (densities < 3 g·cm^−3^), low synthesis temperature (< 1500°C) alternative, surpassing the refractive indices (up to ∼2.1) and elastic moduli (up to ∼100 GPa) of oxide glasses with comparable cationic matrices (as shown in Figure 7e–h) [3, 38, 89]. Notably, glass‐forming nitride compositions in this system are relatively P^5+^‐poor (i.e., depolymerized, Li^+^‐rich matrices) with respect to oxide matrices, suggesting enhanced ionic transport, and thus relevance as a grain‐boundary‐free solid‐state electrolyte. Although Li_3_N and P_3_N_5_ are not included in the SpMCBN database of the FactSage software suite–thereby preventing the quantitative evaluation of liquidus temperatures in the P‐based system–, the approximately 70 experiments (successful, or not) from the original study identified that these ternary all‐nitride batches melt at temperatures below 1500°C [20]. In fact, such low synthesis temperatures–anticipated from the notably small ambient‐pressure melting temperatures of Li_3_N (813°C [246]) and Ca_3_N_2_ (1195°C [247]); noting that P_3_N_5_ decomposes prior to melting at ambient pressure–suggest that P‐based nitride glasses represent the most suitable systems for stabilizing metal nitrides prone to high‐temperature decomposition (e.g., Fe_4_N, Mn_6_N_5_). Conversely, B‐based nitride systems (see Figure 7i–l) require higher melting temperatures but offer superior elastic moduli (up to ∼270 GPa) at comparatively very small densities (< 3 g·cm^−3^), and refractive indices exceeding those of heavy‐metal‐containing oxide glasses (up to ∼2.2). These systems collectively illustrate the diversity of achievable properties in nitride glasses–though they do not encompass the full extent of the accessible compositional space–, which are summarized along with key synthesis parameters in Table 2.

**TABLE 2:** Summary of the representative nitride glass systems considered, including key synthesis parameters, associated challenges, and projected properties computed through the present framework. When available, the oxide and nitride glass‐forming domains are provided to reflect the breadth of these regions within the (cationic) ternaries.

Glass chemistries			All‐nitride glasses synthesis parameters			All‐nitride glasses projected properties		
Cationic system	Oxide glass‐forming domain (%)	Nitride glass‐forming domain (%)	Liquidus temperature, T_liq_ (°C)	Minimum N_2_ pressure (GPa) to suppress nitride decomposition (P_atm_ = 10^−4^ GPa)	Technical challenges	Density, ρ (g·cm^−3^)	Elastic modulus, E (GPa)	Refractive index, n (‐)
Y^3+^–Si^4+^–Al^3+^	50	?	1700–2600	10^−4^–5·10^−2^	Requires adapted furnace assemblies (most Pt alloys melt below 1800°C)	3.0–7.3	175–245	1.9–4.1
Li^+^–P^5+^–Ca^2+^	46	12	< 1500	≥ 8·10^−1^	P_3_N_5_ imposes high (GPa scale) N_2_ pressures for suppressing decomposition	1.9–4.1	85–120	1.7–2.8
Ca^2+^–B^3+^–Al^3+^	30	?	1900–2600	10^−4^–3·10^−2^	Most compositions are not meltable using conventional piston‐cylinder apparatuses (T_liq_ > 2300°C)	1.9–4.1	85–275	1.7–2.7

### 3.4 Outlooks and Outcomes

In this final section, perspectives are outlined regarding the synthesis and handling of all‐nitride glasses, as well as their scalability, processing, and potential avenues for advanced characterization and application‐oriented case studies. Furthermore, the role of these innovative materials–exhibiting mixed ionic, covalent, and metallic (when nitrogen is located in interstitial sites) bonding–is emphasized in advancing the fundamental understanding of the glass transition, while simultaneously fueling the exploration of glass‐forming systems with previously uncharted chemistries. A general workflow to this end is illustrated in Figure 8.

**FIGURE 8:**
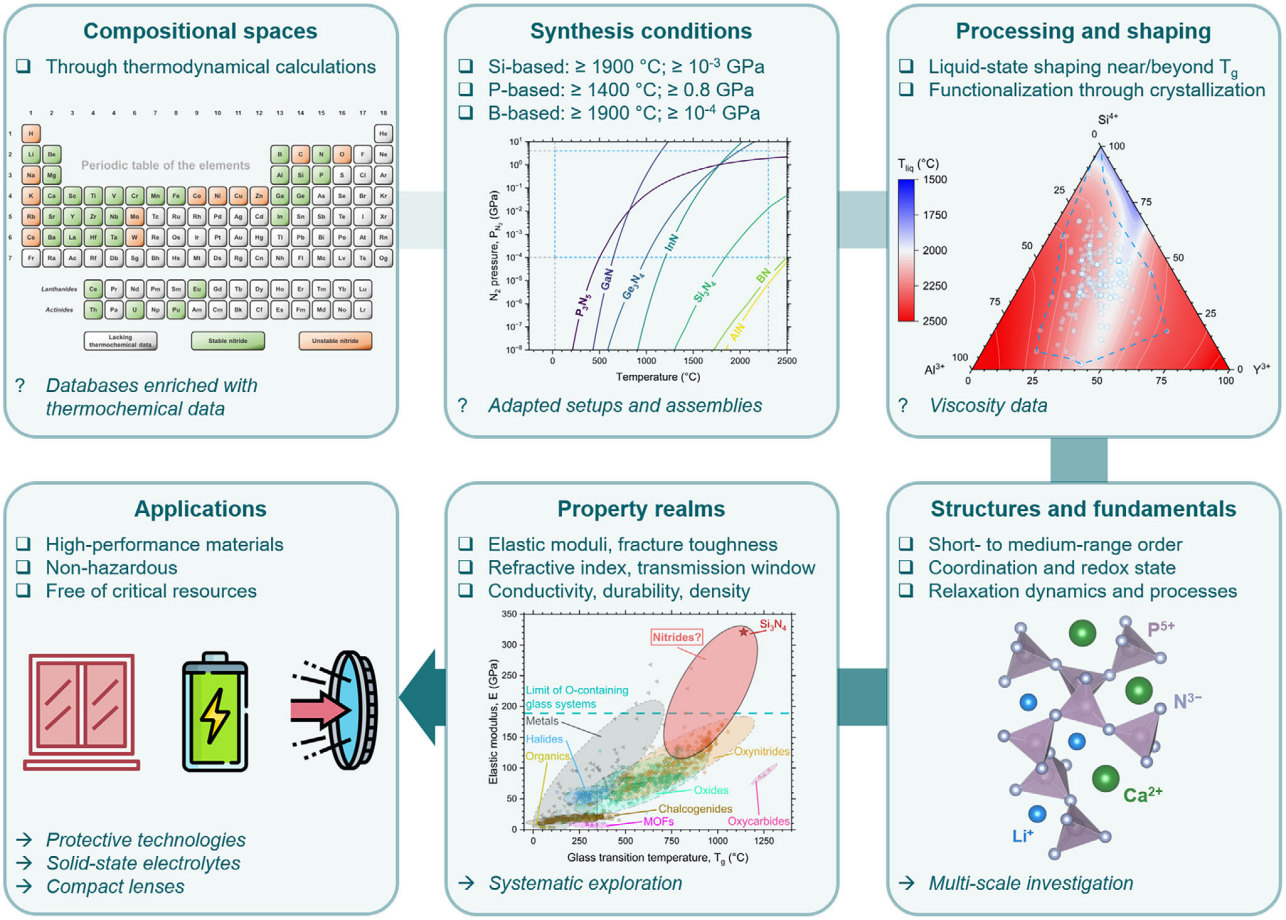
General workflow for the design, synthesis, and processing of all‐nitride glasses, with subsequent investigation of their structure, properties, and prospective applications.

#### 3.4.1 Synthesis Pressures, Sample Size, and Raw Materials

Based on the thermodynamical findings presented in Section 3.1 and Section 3.3, wherein the temperature and pressure conditions required to achieve melting without decomposition were identified, the attainable laboratory‐scale size of bulk, homogeneous P‐based nitride glasses appears to be limited, under current synthesis technologies, to approximately the cm^3^ scale for conventional piston‐cylinder apparatuses. Nevertheless, should their exceptional properties at this scale be confirmed through preliminary experimental validation, the development of tailored engineering solutions targeting these specific conditions may enable further scaling. In contrast, Si‐ and particularly B‐based nitride glasses demonstrate greater scalability. For instance, boron nitride (BN), when processed under a nitrogen atmosphere, exhibits no decomposition at ambient pressure up to 2500°C, whereas the pressures required to suppress the decomposition of silicon nitride (Si_3_N_4_) are orders of magnitude smaller than those necessary for phosphorus nitride (P_3_N_5_). Consequently, the fabrication of such high‐mechanical‐performance systems does not require ultrahigh pressures–typically associated with diamond anvil cells (DACs), which restrict samples to the µm scale; notwithstanding the exceptionally high elastic moduli reported for such specimens (> 450 GPa for densified a‐SiO_2_ [248, 249])–, thereby unlocking scalable fabrication at cm^3^ dimensions and potentially beyond. Nevertheless, recent advances in DAC design and accessibility offer opportunities for the in situ exploration of these original systems [250, 251]. In parallel, ongoing progress in the development of high‐purity nitride precursors is particularly timely and critical for advancing the synthesis of nitride glasses [252, 253, 254, 255].

#### 3.4.2 Temperature, Viscosity, and Crucible Material for Melting

The primary bottleneck to scaling Si‐ and B‐based nitride glasses lies in the high temperatures required not only for melting but also for achieving homogeneity. This challenge may be partially addressed through (i) optimized powder preparation techniques, such as solvent‐assisted milling, and (ii) modification of the chemical composition by identifying deep eutectics, perhaps beyond ternary systems and towards quaternaries and more. The latter could subsequently reduce the pressure required for synthesis, thereby enabling the use of alternative methods such as hot pressing or high‐quenching‐rate techniques, including spark plasma sintering (SPS). Furthermore, although piston‐cylinder apparatuses were first established over fifty years ago, critical advances continue to emerge, more than three decades after the seminal study on all‐nitride glasses. These developments are particularly relevant to the hypothetical nitride glass systems discussed in Section 3.3: they are poised to enhance accessibility to their (extreme) synthesis and facilitate scalability–beyond the 50 mg scale achieved in the original work [20]. Key innovations include piston‐cylinder systems capable of reaching temperatures up to 3000 K [256], but also quenching rates of up to 160 K·s^−1^ [257], improved operability and precision [258], a wide breadth of case‐study‐specific furnace assemblies [259], more homogeneous temperature distribution [260], and low‐pressure capabilities (down to 150 MPa) [229, 261]. An additional critical factor in nitride glass synthesis is the consideration of their viscosity and glass transition temperature. Although quantitative viscosity data remain unavailable, qualitative values were reported for the P‐based nitride glasses of the original study [20]. In fact, several (though not all) glass‐forming systems exhibit a ratio of melting temperature to glass transition temperature (T_m_/T_g_ in K/K) of approximately 2/3 [262]. For Si_3_N_4_, assuming a T_g_ of 1140°C (deduced from the temperature dependence of its elastic modulus [37]) and an ambient pressure melting temperature near 1800°C (as extrapolated from the pressure dependence of its melting temperature [221]), a close value of 0.68 is obtained, comparable to that of fused silica (0.73). Such a T_g_ suggests that, even under conservative assumptions of strong liquid behavior (as suggested in the original study [20]; e.g., m = 15) [263], viscosities suitable for casting (∼10^4^ Pa·s at 2300°C) are achievable [245]. However, further development would necessitate modifications to the original furnace assembly, notably as platinum–employed as the outer encapsulating material in the double encapsulation approach–becomes unsuitable above ∼1700°C. Combined with the observation that nitride glass formation requires only conventional cooling rates of ∼100 K·s^−1^ during melt‐quenching [264], and that high pressure serves solely to suppress nitride decomposition, these considerations underscore the potential of nitride glasses as a promising and accessible class of functional materials for processing and application. It is worth noting that such strategies may enable the production of long‐sought transparent yet stiff, nitrogen‐rich, Si‐based oxynitride glasses, as well as hypothetical glassy Si_3_N_4_, thereby complementing existing studies of amorphous silicon nitride [265].

#### 3.4.3 Handling and Processing

Post‐synthesis, P‐based nitride glasses have been reported to remain stable under air [20]. Furthermore, the observed decrease in dissolution rates by more than three orders of magnitude with increasing nitrogen substitution in oxynitride glasses (at constant cationic composition) indicates a high resistance to corrosion for all‐nitride glasses [266]. As a result, such materials are expected to be amenable to polishing in water or in other solvents such as ethanol. Although not investigated, surface oxidation, commonly encountered with nitride materials [267], may occur. In addition, while the thermal stability parameter (ΔT = T_x_ − T_g_, where T_x_ denotes the onset of crystallization) was not quantified, the observation of viscous flow at ambient pressure and their apparent strong character suggests that liquid‐state shaping is feasible, provided that a protective atmosphere is maintained (e.g., heating in flowing nitrogen gas or in a glove box with a controlled atmosphere [8, 268]). This behavior is likely associated with the kinetically hindered decomposition of the constituent nitrides, despite thermodynamic predictions indicating, for instance, decomposition of P_3_N_5_ under such conditions (see Section 3.1). It also implies that controlled crystallization may be achieved using conventional glass‐processing methodologies. Finally, based on analogous behavior in oxynitride systems, nitride glasses are expected to exhibit resistance to bulk oxidation in air up to temperatures approaching T_g_ [269].

#### 3.4.4 Functionalization Pathways and Applicability Prospects

The suitable handling and formability of nitride glasses enable not only the fundamental characterization of their structure and properties but also their manufacturing and deployment in high‐performance applications (i.e., requiring elevated merit indices). A direct application can be envisaged in the form of high‐refractive‐index, mechanically stiff, and compact lightweight optical components, such as lenses, exhibiting broad and tunable transmission windows extending from the visible to at least the near‐infrared. Such materials are, however, anticipated to exhibit relatively small Abbe numbers [192, 270]. Key parameters, including band gap, phonon energies, and optical losses–critical for electro‐optical and information technologies [271]–remain to be determined for all‐nitride glasses. Established glass functionalization strategies, including doping with redox active species, controlled crystallization, and ion‐exchange processes, may further widen their range of possibilities (stress‐induced luminescence, piezoelectricity) and accessible properties (opto‐magnetic response, high ionic conductivity, enhanced fracture toughness) [272, 273, 274]. All in all, refractory glass‐containing nitride systems present a compelling class of materials with exceptional properties and the potential to serve as critical‐resource‐free, non‐hazardous alternatives to established glasses (e.g., TeO_2_‐, Ge‐, AlF_3_‐, or rare‐earth‐rich systems).

#### 3.4.5 Contributions to the Fundamentals of the Glass Transition

All‐nitride glasses constitute, in contrast to all previously known glass families, a unique system wherein chemical bonds within the glass network involve a blend of ionic, covalent, and metallic character [275, 276, 277, 278]–with the additional possibility, under high‐pressure conditions, of forming infinite N–N chains [279, 280]. Beyond their broad potential within materials science, this mixed bonding provides an exceptional model system for advancing the fundamental understanding of glass formation and the glass transition, offering, notably, the prospect to unify concepts across metallic and non‐metallic glass‐forming systems. Achieving such insight will require a comprehensive set of investigations, particularly in situ, employing a battery of well‐suited spectroscopic techniques to probe the topology, connectivity, and bonding of all‐nitride glasses, and of the liquids from which they derive. Emphasis should be placed, in a first stage, on elucidating both short‐ and medium‐range order, as well as the coordination environments and redox states of constituent elements. Established methods–including Raman spectroscopy, solid‐state nuclear magnetic resonance spectroscopy (^15^N, ^29^Si, ^31^P), Mössbauer spectroscopy (e.g., ^57^Fe) and X‐ray photoelectron spectroscopy–will be instrumental, supported by the extensive existing literature on nitrogen‐containing thin films and bulk systems. Such approaches are expected to yield an atomic‐scale description of all‐nitride glasses, thereby informing their modeling and rational design. From a broader perspective, it remains uncertain whether pressure‐driven synthesis strategies could enable the formation of hypothetical boride, carbide, or phosphide glasses, which are among the few inorganic, non‐metallic systems for which glass formation has not been demonstrated, yet could offer superior performance [281, 282, 283, 284, 285].

## 4 Conclusions

In this work, all‐nitride glasses were reconsidered within the broader context of established and emerging glass families. Drawing on evidence from the original study reported three decades ago, together with compositional trends accumulated over more than sixty years of research on oxynitride systems, nitride glasses were identified as a promising class of materials. In fact, their synthesis has now become increasingly accessible owing to recent advances in raw material purity and high‐pressure synthesis technologies, thereby providing a pathway for achieving optically transparent glasses with access to a superior property space.

To substantiate this perspective, conventional approaches in glass science were employed to estimate key properties, including the use of the Makishima–Mackenzie model for elastic moduli, but also complemented by an inverse application of the optical basicity concept for refractive index prediction. Following validation across both glassy and crystalline systems, encompassing oxide and nitride chemistries, these methods were applied to delineate (through semi‐empirical computations) the accessible property realm of all‐nitride glasses.

When combined with thermodynamic strategies, this framework enabled the estimation of attainable compositional domains and corresponding liquidus temperatures compatible with synthesis using conventional piston‐cylinder apparatuses. This approach was demonstrated for three representative systems–Si‐, P‐, and B‐based–reflecting archetypal compositions extensively studied in oxide glass science. The resulting predictions identified advantageous systems exhibiting unique combinations of large elastic moduli and refractive indices at comparatively small densities. Such performance, achieved without reliance on critical or hazardous raw resources, underscores their capability for high‐performance applications, further extendable through well‐established processing and functionalization routes.

Although these projections highlight some exciting perspectives, systematic experimental and further theoretical investigations into all‐nitride glasses and their parent liquids remain essential. Significant knowledge gaps must be covered regarding synthesis parameters, high‐temperature behavior, scaling, structural organization, and overarching macroscopic properties.

Finally, it is hypothesized that fundamental studies of these systems could contribute to advancing the understanding of glass formation and the glass transition, as they uniquely combine ionic, covalent, and metallic bonding. Whether similar approaches may be extended to further broaden the range of glass‐forming systems, for instance towards hypothetical boride, phosphide, or carbide glasses, remains an open question.

## Author Contributions

**Alexis Duval**: Writing – original draft, Methodology, Investigation, Data curation, Conceptualization. **Tor Grande**: Writing – review & editing. **Lothar Wondraczek**: Writing – review & editing, Conceptualization, Methodology, Supervision.

## Conflicts of Interest

The authors declare no conflicts of interest.

## Supporting information




**Supporting File 1**: advs77963‐sup‐0001‐TableS1.xlsx.

**Supporting File 2**: advs77963‐sup‐0002‐TableS2.xlsx.

**Supporting File 3**: advs77963‐sup‐0003‐TableS3.xlsx.

## Data Availability

All data supporting the findings in this study are available within this Article and its Supplementary Materials. Any additional data are available from the corresponding authors upon request.
